# Food allergy and anaphylaxis – 2045. Highly elevated IgE antibodies to vaccine components in influenza vaccine-associated anaphylaxis in Japan

**DOI:** 10.1186/1939-4551-6-S1-P130

**Published:** 2013-04-23

**Authors:** Mizuho Nagao, Takao Fujisawa, Toshiaki Ihara

**Affiliations:** 1 Institute for Clinical Research, Mie National Hosipital, Japan

## Background

Anaphylaxis after vaccination is rare but significant problem since it may be fatal without prompt treatment. Proper diagnosis and identification of the causative agent is critical for management and prophylaxis of the anaphylaxis. Influenza vaccine-associated anaphylaxis (IVA) has been related to egg allergy since influenza vaccines are produced in embryonated eggs. However, patients with IVA do not always have egg allergy. In 2011-12 season, increase in incidence of IVA was reported from one manufacturer in Japan (approximately, 1 in 1.4 million doses in regular years and 1 in 0.4 million doses in 2011).

## Objectives

To identify the cause of the anaphylaxis events in 2011-12 in Japan.

## Methods

We collected serum and blood specimens of IVA cases within 2 months after the events from all areas of Japan. The diagnosis was confirmed based on the Brighton collaboration case definition of anaphylaxis of level 1 and 2. Eighteen cases of confirmed IVA and age-matched 7 control subjects with the similar vaccination history were examined. Specific IgE to each component, namely A/H1, A/H3 and B, of the trivalent vaccines distributed for 2011-12 season from several vaccine manufacturers was measured with ELISA. Antigen-induced basophil activation was evaluated by measurement of CD203c expression with flowcytometry. Effects of additives to the vaccine preparations on the CD203c expression were also examined.

## Results

No patients with IVA had egg allergy. Specific-IgE antibodies to A/H1, A/H3 and B were significantly elevated in patients with IVA than in controls. No differences in IgE antibody titers among components or products from different manufacturers were identified. Influenza vaccine component-induced CD203c expression in basophils were also highly enhanced in IVA and no response was observed in controls. Since the IVA cases segregated in patients who received phenoxyethanol-containing vaccines, effect of the preservative on basophil activation was examined and enhancement with phenoxyethanol, not with thimerosal, of the response was observed in some cases.

## Conclusions

The results suggest that the recent IVA in Japan was caused by specific IgE antibodies to influenza vaccine components and that phenoxyethanol may have modified the reaction. Measurement of vaccine-component-specific IgE and basophil activation is useful for diagnosis of vaccine-associated anaphylaxis.

